# Functional characterization of the late embryogenesis abundant (LEA) protein gene family from *Pinus tabuliformis* (Pinaceae) in *Escherichia coli*

**DOI:** 10.1038/srep19467

**Published:** 2016-01-19

**Authors:** Jie Gao, Ting Lan

**Affiliations:** 1 Key Laboratory of Tropical Forest Ecology, Xishuangbanna Tropical Botanical Garden, Chinese Academy of Sciences, Menglun, Yunnan, China; 2 State Key Laboratory of Systematic and Evolutionary Botany, Institute of Botany, Chinese Academy of Sciences, Beijing 10093, China

## Abstract

Late embryogenesis abundant (LEA) proteins are a large and highly diverse gene family present in a wide range of plant species. LEAs are proposed to play a role in various stress tolerance responses. Our study represents the first-ever survey of LEA proteins and their encoding genes in a widely distributed pine (*Pinus tabuliformis*) in China. Twenty–three *LEA* genes were identified from the *P. tabuliformis* belonging to seven groups. Proteins with repeated motifs are an important feature specific to *LEA* groups. Ten of 23 pine *LEA* genes were selectively expressed in specific tissues, and showed expression divergence within each group. In addition, we selected 13 genes representing each group and introduced theses genes into *Escherichia coli* to assess the protective function of PtaLEA under heat and salt stresses. Compared with control cells, the *E. coli* cells expressing PtaLEA fusion protein exhibited enhanced salt and heat resistance and viability, indicating the protein may play a protective role in cells under stress conditions. Furthermore, among these enhanced tolerance genes, a certain extent of function divergence appeared within a gene group as well as between gene groups, suggesting potential functional diversity of this gene family in conifers.

Abiotic stresses, such as drought, salinity, and extreme temperature are limiting factors for normal plant growth and development. High salinity upsets homeostasis in water potential and ion distribution at both cellular and whole plant levels^1^. It results in molecular damages, arrest of development and even mortality^2^. High temperature can cause irreversible damages including protein denaturation, aggregation and degradation^3^, as well as protein synthesis inhibition and enzymes inactivation. Extreme temperature can also increase fluidity of membrane lipids^4^ which could lead to loss of membrane integrity^5^,^6^,^7^. Higher plants have developed several genetic responses to these environmental factors^8^. An important group is the late embryogenesis abundant (*LEA*) protein gene family which functions in cellular protection during abiotic stress tolerance^9^,^10^. Since the first discovery of the LEA proteins in cotton and wheat^11^,^12^, the LEA proteins have been described throughout the entire plant kingdom, as well as in other organisms, from invertebrate to prokaryotes^13^,^14^,^15^.

The *LEA* genes are mainly expressed in seeds and, as its name suggests, are accumulated during late embryo development stage, comprising up to 4% of cellular proteins^16^. They have also been found in seedlings, roots and other organs throughout the whole developmental stage. After suffering environmental stresses, plants accumulate high levels of LEA proteins in response^17^,^18^. They have been proposed to have various functions, including protection of cellular structures from the effects of water loss and desiccation^19^, protection of proteins from stress-induced damage^19^,^20^,^21^, sequestration of ions^20^, and folding of denatured proteins^22^. LEA proteins can also act as chaperone proteins to resist cellular damage^18^,^23^.

The *LEA* genes often exist as a large gene family in higher plants. For example, *Arabidopsis* and *Populus* contain 51 and 53 members respectively, which are divided into eight groups based on the amino acid sequence homology and specific motifs^24^. To date, comprehensive analysis of LEA genes have only been made in angiosperms like *Arabidopsis thaliana*^25^, *Oryza sativa*^26^, *Hordeum vulgare*^27^, *Prunus mume*^28^, *Populus*^24^, *Solanum*^29^,^30^, *Malus domestica*^31^ and *legumes*^32^. In contrast, considerably fewer *LEA* genes have been identified in gymnosperms, mostly due to limited genome information. Only a few *LEA*s and *LEA-like* proteins have been examined in *Picea glauca*^33^, *Pseudotsuga menziesii*^34^ and *Pinus pinaster*^35^.

*Pinus tabuliformis*, a dominant conifer species is widely distributed over mountainous areas from northern to central China. It grows from 0–2700 m above sea level. Most of the region has heterogeneous habitats, including a wide range of temperatures, altitudes, elevated ozone and UV levels^36^. Long generation time and large effective population sizes of *P. tabuliformis* suggest that this species has adapted to variable environmental conditions. Gymnosperms, especially conifers represent a large group of plants with a long evolutionary history. However, long generation time makes it a challenge to investigate transgenic functioning in conifers. The accumulation of *LEAs* during environmental stresses also makes the study of a single LEA gene difficult. Therefore, an alternative approach is to express the PtaLEA proteins *in vivo* in yeast or *E.coli* for functional expression screening, which has been successful in previous studies^37^,^38^,^39^,^40^,^41^. By integrating phylogenetic analysis, protein motif structure, gene expression and heterologous expression analysis in *E. coli* of 23 full-length LEA genes, we examine the evolutionary and functional diversity of the LEA gene family in *P. tabuliformis*.

## Materials and Methods

### Identification of LEA genes from the *Pinus taede* EST database

We identified LEA genes from *Pinus taeda* by searching 53 *Arabidopsis* LEA protein sequences in the *P. taeda* EST database (328,662 ESTs in the National Center for Biotechnology Information database: http://www.ncbi.nlm.nih.gov/) using TBLASTN. All the identified *P. taeda* candidates were analyzed using the protein family database (Pfam; http://pfam.sanger.ac.uk/) to confirm the presence of *LEA* conserved domains in their protein structure. We retrieved 43 whole *LEA* genes from *P. taeda*, allowing us to design 43 primers based on these gene sequences and use them to clone the *LEA* genes from *P. tabuliformis*.

### Molecular cloning, phylogenetic analysis and bioinformatic analysis of *Pinus tabuliformis* LEA genes

Total RNA isolation from seeds of *P. tabuliformis* was performed using an Aurum Total RNA Kit (Bio-Rad, Hercules, CA, USA). Total RNA was treated with RNase-free DNase I (Promega, Madison, WI, USA) and the first strand cDNA was synthesized using an RNA PCR Kit (AMV) version 3.0 (TaKaRa, Otsu, Japan). PCR conditions consisted of an initial denaturation of 3 min at 94 °C, followed by 35 cycles of 30 s at 94 °C, 40 s at 55 °C and 60 s at 72 °C, and a final extension of 5 min at 72 °C. PCR products were cloned into *pEASY*-T3 (TransGen, Beijing, China), and sequenced in both directions to verify the gene sequence. We confirmed that the sequences obtained had LEA conserved domains using Pfam.

We cloned 23 full-length *LEA* genes from *P. tabuliformis*. The multiple sequence alignment of the 23 *PtaLEA* genes was performed using MUSCLE^42^ (http://www.ebi.ac.uk/Tools/msa/muscle/) with default parameters and manually adjusted by BioEdit^43^. Phylogenetic analysis was carried out by the maximum parsimony method with 1000 bootstrap replicates using MEGA v.5 software^44^.

Conserved motifs for each LEA proteins were investigated using the Multiple Expectation maximization for Motif Elucidation (MEME) system version 4.10.24^45^ (http://meme.sdsc.edu/meme/cgi-bin/meme.cgi), any number of repetitions are expect to be distributed in sequences, with the minimum width of 6, and maximum width of 50. The signal peptide analysis was done by the TargetP algorithm^46^ (TargetP: http://www.cbs.dtu.dk/services/TargetP/).

### Transcription abundance of PtaLEA genes in *P. tabuliformis* tissues

To determine the expression pattern of each *P. tabuliformis LEA* gene, specific primers of the 23 *LEA* genes were designed (Table S-2) and used in RT-PCR, based on the multiple sequence alignment of *LEA* gene sequences. Total RNA was isolated from the top bud, needle, phloem in the stem, root of more than 10-year old *P. tabuliformis* and also two weeks germinants. Total RNA was treated as in the previously described method. Amplification was performed in a volume of 25 μL containing 10 pmol of each primer, 2.5 μL TaKaRa 10 × PCR buffer, 0.125 μL TaKaRa ExTaq (5 U/μL), 2 μL dNTP (2.5 mM each). PCR conditions consisted of an initial denaturation of 3 min at 94 °C, followed by 25 cycles of 30 s at 94 °C, 40 s at 60 °C and 1 min at 72 °C with a final extension of 3 min at 72 °C. In all PCR analysis, the *Actin* gene was used as an internal control. The PCR products were analyzed by 1.5% agarose gel electrophoresis. PCR products from each sample were validated by DNA sequencing. For each biological sample three repetitions were performed.

### Construction of *E.coli* strains expressing LEA proteins

First, we added a NotI site upstream from the start codon ATG with the 5′ end primer, and a SpeI site after the stop codon TAA with the 3′end primer. Then, the LEA genes we cloned from the first step were used to do the PCR amplification. The PCR products were ligated into T-vector (Promega Madison, USA). The vector was then digested using a NotI/SpeI double digestion, and the resulting DNA was gel-purified and subcloned into the TWIN1 vector (New England BioLabs, Beijing, China), linearized by a double digestion with the same restriction enzymes to construct TWIN-LEA plasmid. The *PtaLEA* coding region and the junction sequences were confirmed by DNA sequencing. The TWIN-LEA plasmid was transformed into applicable *E. coli* host strain BL21 (DE3). Transformed *E. coli* cells were grown in Luria–Bertani (LB) medium containing 100 mg /ml of carbenicillin and kept at 37 °C overnight. The overnight cultures were diluted 1,000-fold using fresh LB medium and incubation continued at 37 °C until mid-log phase (3–4 h, OD_600_ = 0.6). Isopropyl b-D-thiogalactopyranoside (IPTG) was then added to a final concentration of 1 mM, and incubation was continued at 37 °C for 3 h. Cells were harvested by centrifugation and resuspended in Buffer 1 [20 mM Tris–HCl (pH 8.5), 500 mM NaCl, 1 mM EDTA] and lysed by sonication. The lysate was centrifuged at 12,000 rpm for 10 min to collect clarified cell extract for SDS–PAGE analysis.

### Heat and salt treatment with transformed *E.coli* cells

Cell cultures were grown as described above. IPTG was added to mid-log phase cultures (OD_600_ = 0.6) to a final concentration of 1 mM, and incubation was continued at 37 °C for 3 h. After IPTG induction, the cultures were diluted and transferred to 50 °C. Samples (10 μl) were taken at 0, 30, 60, 120, and 180 min, and serial dilutions were plated onto LB plus carbenicillin plates. We have detected the values of OD_600_ from each sample and make them to be the same value. Cell viability was estimated by counting the number of colony-forming units after incubation of the plate over- night at 37 °C. For salt treatment, after IPTG induction, ten microliters of each sample was spotted onto the LB plates(and we also controlled the OD _600_ for each of these samples),containing 171 mM NaCl and the LB plates with 300 mM NaCl, 400 mM NaCl, 500 mM NaCl, 600 mM NaCl, 700 mM NaCl, respectively. Plates were then incubated at 37 °C over night.

The colony number on each plate was recorded using the software of Cellprofiler^47^. Viability ratio of the transformants under salt and heat conditions was calculated according to the following formula:
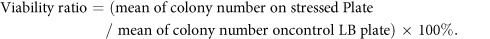


For both treatments (heat and salt), the means of three experiments were determined from three independent transformants.

## Results

### Sequence and structure characteristics of the PtaLEA gene

**Figure 1:**
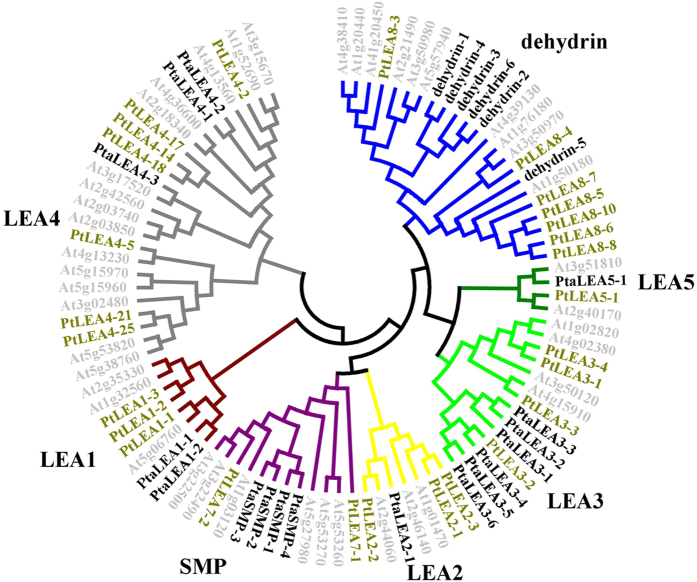
Phylogenetic relationships of *Pinus tabuliformis*, *Populus* and *Arabidopsis LEA* genes. LEA groups are distinguished by color. The light grey represented the LEA genes form *Arabidopsis*, the bold names represented the LEA genes from *P. tabuliformis*, and the green names are genes from *Populus*.

Forty-three full-length genes encoding putative LEA proteins in *P. taeda* were identified from the *Pinus taeda* EST database. Due to the lack of *Pinus* whole-genome sequence information, we designed the primers based on the sequences of *P. taeda*. The *P. tabuliformis* may have the same or even more members of LEA proteins but we successfully cloned 23 full length *LEA* genes from the *P. tabuliformis*. All of these genes had *LEA* conserved domains which was confirmed by the Pfam domain analysis. A phylogenetic analysis based on amino acid sequences of the predicted proteins revealed that the 23 *PtaLEA* genes grouped into seven groups together with the *Arabidopisis* and *Populus LEAs*, confirming that they belonged to the *LEA* family (Fig. 1). We followed the *LEA* gene family nomenclature in Hundertmank and Hincha (2008) and the Pfam domain name. We found that group *LEA3* and *dehydrin*, each have six genes. The *LEA1* group has two members, *LEA4* has three members, and *SMP* has four members, whereas groups *LEA2* and *LEA5* each contained only one gene. We did not obtain any members from *LEA6* group.

**Figure 2:**
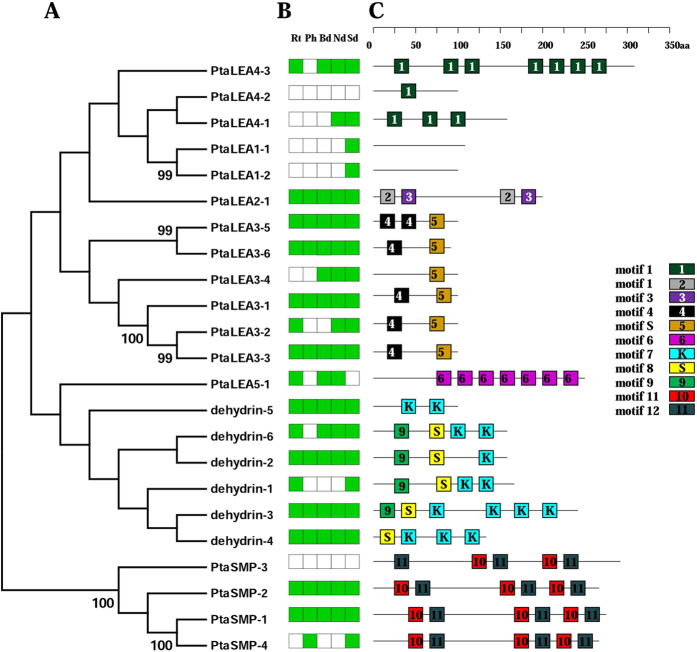
Phylogenetic relationships (**A**), expression patterns (**B**) and motif structure of *P. tabuliformis LEAs*. In B, the green box indicates positive detection of gene expression in the corresponding tissue under root (Rt), phloem (Ph), bud (Bd), needle (Nd), two weeks germinants (Ge). In C, boxes labeled with numbers are protein motifs; the motif sequences are provided in the supplemental Fig. 1.

**Figure 3:**
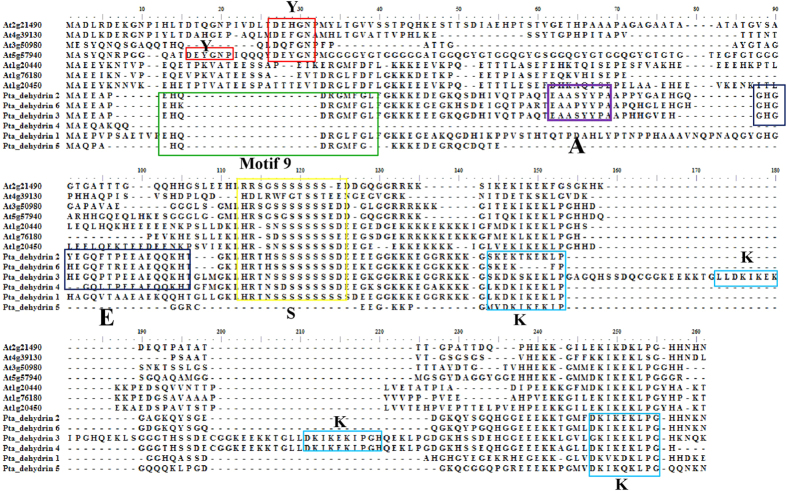
Alignment of the amino-acid sequences of *P. tabuliformis* dehydrins and previously known *dehydrin* genes from *Arabidopsis*. Alignments were performed using the CLUSTAL v program. Dashes indicate where a sequence has been expanded to allow optimal sequence alignment. The Y-, S-, K-, A- and E-segments are indicated by color boxes.

These genes and their families possessed several group-specific characteristics (Table 1; Fig. 2). A majority of *P. tabuliformis* LEA proteins contained repeated motifs that were often specific to a LEA group. We analyze the group-specific characteristics with the comparison to the conserved motifs indentified in *Arabidopsis*^48^ and other species^49^,^50^. The LEA4 group had several repeats of conserved motif 1: three times in *PtaLEA4-1*, up to seven times in *PtaLEA4-3* but only once in *PtaLEA4-2*. The PtaLEA5-1 protein from group *LEA5* had the repeated group-specific motif 6 with seven repetitions. Group *dehydrin* is the second largest group in *LEA* family of which most gene members have been cloned in *P. tabuliformis* (6 members: *dehydrin-1/2/3/4/5/6*). MEME analysis showed that the motif structure was variable in this group. All of the six genes share the similar motifs S and K which are the conserved motif characteristic to *dehydrin* proteins, but we failed to find the Y motif in *P. tabuliformis* which could be found in *Arabidopsis* (see the alignment in Fig. 3). Apart from that, the repetitions of the motif K are variable in different *dehydrin* proteins: twice in *dehydrin-5/6/1*, three times in *dehydrin-4*, and up to four times in *dehydrin-3*, but only appeared once in *dehydrin-2*. The variation in motif structure indicates functional divergence.

Computational prediction of the subcellular distribution of the LEA proteins using targetP indicates further differences between the LEA groups. Whereas the members of the most groups are localized in the cytosol (“other” in Table 1 indicates that no signal peptide was detected), the LEA3 proteins are exclusively targeted to mitochondria, and the LEA5 is probably targeted to chloroplasts.

### Transcription abundance of LEA genes in *P. tabuliformis*

In order to get a better understanding of the expression pattern of *P*. *tabuliformis LEA* genes, five different tissues/organs (root, phloem, needle, bud and two weeks germinants; Fig. 1B) were collected and analyzed using RT-PCR to detect the transcription abundance. Among the 23 *PtaLEA* genes, 11 (47.8%) genes were expressed in all tissues/organs examined under normal condition, indicating that *LEA* gene family has a wide distribution and expression in *P. tabuliformis*, and are undoubtedly involved in normal plant growth and development. All the genes (except *PtaLEA4-2, PtaLEA5-1* and *PtaSMP-3*) are expressed in the analyzed germinants, indicating that *LEA* genes have played an indispensable role in the seedling development process of *P. tabuliformis*. Two genes (*PtaLEA4-2* and *PtaSMP-3*) were not expressed in any tissue we studied, possibly because they are expressed at sub-detectable levels, or they are only induced in response to treatments and/or in tissues not including in our study (like seed). The other 10 *LEA* genes were expressed selectively in specific tissues and showed expression divergence within each group. For example, in the *dehydrin* group, the genes *PtaLEA 8-2/3/4/5* were expressed in all tissues, while *dehydrin-6* was expressed in all tissues except the phloem, and *dehydrin-1* was only expressed in the root and germinants. Within LEA3 group, the *PtaLEA3-1/2/5/6* were expressed in all tissues, while *PtaLEA3-3* was expressed in root, needle and germinants, and the *PtaLEA3-4* was expressed in bud, needle and germinants. In group *LEA4*, the *PtaLEA4-1* was expressed in needle and germinants, while the *PtaLEA4-3* was expressed in all tissues except phloem, however the *PtaLEA4-2* did not have detectable expression in any organs. Generally, the different expression pattern of LEA family probably implies the divergence of function between genes within groups.

### Enhancement of heat and salt-tolerance of recombinant *E.coli* cells with PtaLEA genes

In order to determine the function of expressed LEA fusion protein in stress conditions, we selected 2–3 genes from each group except group LEA 2 and LEA5 which we only have one gene in these group (13 genes in total) to construct the recombinant BL/LEA to do the heat and salt treatment.

**Figure 4:**
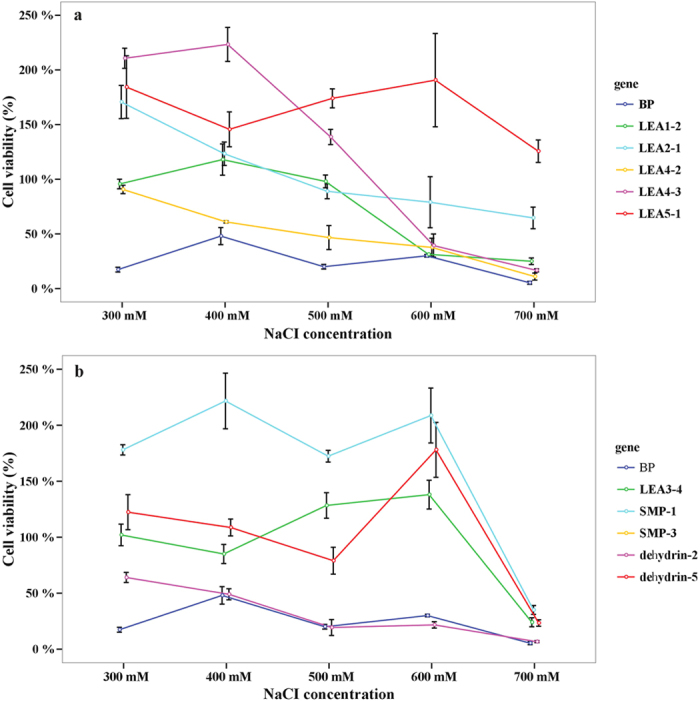
Cell Viability ratio of *E.coli* transformed with TWIN–LEA and TWIN1 constructs under salt treatment. The values are the mean ± SE from three samples.

For the salt treatment, we considered the gene as improving salt tolerance when the viability ratio was higher than the control strain BL/PTWN. The results showed that ten of the thirteen *PtaLEA* genes had mean viability ratios 2–7 fold higher than those of the control strain BL/PTWN under different concentration of salt stress (Fig. 4a,b). These ten genes belong to different groups. We selected *PtaLEA3-3/4*, *PtaLEA4-2/3* and *PtaSMP-1/3* from each group. All these genes, except *PtaLEA3-3*, have shown an increased salt tolerance. For the group *dehydrin*, we detected three genes *dehydrin-4/5/2*, two of which (*dehydrin-5/2*) have shown an increased salt tolerance with a mean viability ratio 4.3 and 1.3 fold higher than the control strain respectively. LEA2 and LEA5 have only one gene in each group, and LEA1 has two members. We chose all members in these three groups for experiments. The results showed that all of them increased salt tolerance except *PtaLEA1-1*. The genes *PtaSMP-1* (6.8 fold), *PtaLEA2-1* (3 fold), *PtaLEA4-3* (5.3 fold), *PtaLEA5-1* (6.8 fold), *dehydrin-5* (4.3 fold) had significantly higher survival ratios than other genes under all concentrations. Genes within group showed different patterns to salt stress, i.e. the viability ratios of *PtaLEA4-3* (5.3 fold), *dehydrin-5* (4.3 fold) and *PtaSMP-1* (6.8 fold) were higher than their group members *PtaLEA4-2* (2.0 fold), *dehydrin-2* (1.3 fold) and *PtaSMP-3* (1.6 fold) respectively.

**Figure 5:**
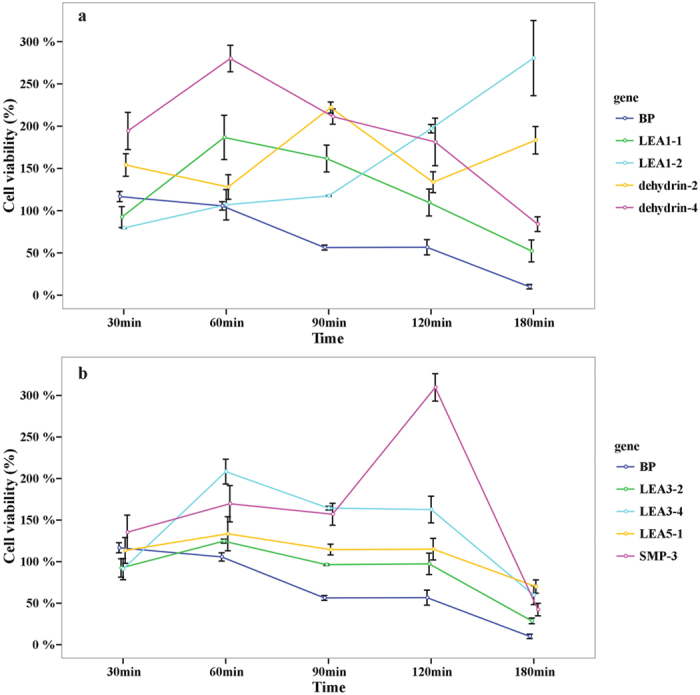
Cell viability ratio of *E.coli* transformed with TWIN–LEA and TWIN1 constructs under heat treatment at the temperature of 50 °C. The values are the mean ± SE from three samples.

Results in the heat treatment demonstrated that seven of the thirteen genes showed signs of increased heat tolerance, with viability ratios higher than the control strain BL/PTWN (Fig. 5a,b). Each group of *LEA1*, *LEA5* and *SMP* had one gene and group *LEA3* and *dehydrin* had two members. After 50 °C induction for 60 minutes, the viability ratio increased significantly which indicated that the genes need a certain induction time. The *PtaLEA3–4* (2.0 fold), *dehydrin-4/2* (1.5/2.4 fold) and *PtaSMP-3* (2.4 fold) showed the highest viability ratio among these genes. Within *LEA3* group, the viability ratio of *PtaLEA3–4* (2.0 fold) is much higher than *PtaLEA3-2* (1.3 fold).

In conclusion, the expressed *P. tabuliformis* LEA fusion polypeptide conferred salt and heat tolerance to the host cells. Some of the groups showed an increased resistance to heat and salt, but not significantly, like *dehydrin-2* that increased the viability by 1.3 fold under salt stress and *PtaLEA3-2* that increased the viability by 1.3 fold under heat stress. Some of the groups showed responses to both heat and salt stresses, but genes within group showed different patterns. In particular, within group *dehydrin*, the *dehydrin-5* (4.3 fold) showed higher viability under salt treatment, *dehydrin-4* (1.5 fold) showed higher viability under heat treatment, while *dehydrin-2* responded to both heat (2.4 fold) and salt (1.3 fold) treatment. All these results suggested that function divergence appeared not only between groups but also between genes within groups.

## Discussion

The *LEA* genes play crucial roles during embryonic development and response to abiotic stresses throughout the plant kingdom as well as in many other organisms. Our molecular evolution analysis detected 43 gene members in the *Pinus taeda* genome, fewer than in *Populus*^24^ which has 53 members. In this study, we have cloned 23 genes from the *P. tabuliformis* genome belonging to seven groups of the *LEA* gene family, the protein sequence identities among groups is very lower than the values within each group which exhibited a high degree of divergence among the protein groups.

Previously, the functional expression screening of LEA proteins *in vivo* in yeast or *E.coli* under abiotic stresses have been investigated successfully in various flowering plant species. Wang *et al*. (2008) investigated an *LEA* gene from *Tamarix* expressed in yeast which showed enhanced tolerance to high temperature, NaHCO_3_, ultraviolet radiation, salt, drought and freezing. Reportedly, one of *LEA3* proteins (*HVA1*) displayed increased tolerance to salt stress and water deficit in both yeast expression system and transgenic rice plants^51^,^52^. Similarly, one *LEA4* gene from *Brassica napus* is able to enhance the cellular tolerance to temperature and salt stresses of *E. coli* cells^53^. More recently a SMP protein from tea has reported function as a chaperone enhanced tolerance to *E. coli* against stresses^40^. However, only a few LEA proteins increasing the tolerance to abiotic stresses had been studied and were primarily from the major group *LEA3*, *LEA4* and *dehydrin*. No gymnosperm *LEA* genes had been screened prior to this study. Furthermore, there was no study about the molecular mechanism of *P. tabuliformis* adapting to stressful environments. The *P. tabuliformis* has a very large distribution area including high temperate, arid and semiarid regions^36^. The gradual salinization of land and the increased summer forest fires have been identified as the major threats to the pine forest in these regions^54^,^55^,^56^. A study showed that *Pinus pinea* seed germination is sensitive to drought, salinity and heat influence^57^. And another report also confirmed that the reduction of *dehydrin* proteins in seed have reduced seed longevity in *Arabidopsis thaliana*^58^. This might be the same in the *P. tabuliformis* seed germination. The LEAs are considered playing a pivotal role in the late embryonic development during the seed germination^16^. In this study, 70% and 50% of the PtaLEAs analyzed increased the tolerance to salt and heat stresses, respectively. These genes come from almost all groups of the *LEA* gene family, which indicates the whole gene family has a function in stress response. Thus, for the first time we have confirmed that the *P. tabuliformis* LEA proteins from all *LEA* subgroups enhance tolerance to elevated salt and heat conditions. It indicates that the *LEA* genes have a wider repertoire of roles in stress responses and may play a common role in plant acclimation to stress conditions. This could be due to their highly conserved sequence motifs and high content of hydrophilic amino acids^21^, and we will discuss it later. Furthermore, the LEA subgroups represented diversified adaptation to heat and salt stress. *LEA1* increased the resistance to heat and salt, but not significantly, its viability ratio showed 2.3 and 3.1 fold higher than the control strain for heat and salt treatments respectively; while *SMP*, *LEA4* and *LEA5* induced a higher tolerance to salt stress (more than fivefold higher than the control strain), and *LEA3*, *dehydrin* induced a higher tolerance to heat treatment (more than twofold than the control strain). For large gene families with multiple subgroups, genes from different groups usually show functional diversification^59^ Another functional analysis performed using the *E. coli* heterologous expression system, including three genes from group *LEA1*, *LEA3* and *dehydrin* respectively, also supported the hypothesis that genes from *LEA1* and *LEA3* increased the tolerance to salt and low temperature, while genes from *dehydrin* did not induce obvious growth improvement^60^. While a recent study has investigated functions of six groups of LEA proteins from *Arabidopsis thaliana* which were expressed in *Saccharomyces cerevisiae* under desiccation stress. None of proteins from *LEA1*, *LEA5* and *AtM* showed protection but proteins from *LEA2*, *LEA4* and *dehydrin* has enhanced tolerance to desiccation stress^41^.

In addition to the expected functional divergence between gene groups, we observed substantial divergence between genes within a single group. The phenomenon was especially apparent in group *LEA4* and group *dehydrin*. *LEA4* is the largest group in *LEA* gene family, with the *Populus* and *Arabidopsis* genomes containing 26 and 16 members, respectively^24^,^25^. It is reported that this group is very heterogeneous and the proteins differ greatly in size and GRAVY^25^. *LEA4* is also the largest group (16 genes) in *P. taeda*. In *P. tabuliformis* we cloned three members and investigated two of them under stress conditions. Within group *LEA4*, the gene *PtaLEA4-3* induced a higher salt tolerance (5.3 fold) than the gene *PtaLEA4-2* (2.0 fold). Clear divergence in expression patterns and motif structure was observed between the two genes. *PtaLEA4-3* had the group-specific motif 1 repeated up to seven times, and its expression was observed in all of the tissues except phloem. The PtaLEA4-2 had only one repetitions of motif 1 that did not express in any tissue we studied. The enhanced salt-tolerance of *PtaLEA4-3* with more motif repetitions was consistent with a previous study which investigated the repeat region (a highly conserved 22-mer motif) in the PM2 (*LEA3*) protein in *E. coli*. They have found that the recombinant strains with more repeat motifs had much higher viability ratios than the recombinant strains with shortened repeat motifs^61^. This evidence proved the fact that the conserved repeat motifs in LEA protein have played important function in salt tolerance.

The *dehydrin* group, also referred to as *dehydrins*, is the second largest group in the *LEA* family. Within this group, the *dehydrin-5* induced a higher viability ratio to salt treatment, and *dehydrin-4* increased the heat tolerance, while *dehydrin-2* responded to both treatments. The *P. tabuliformis* proteins share the highly conserved motif elements of K-segment and S-segment, but they lack the Y-segement [(V/T) DEYGNP], which consistent with the report that this segment only been described in angiosperm^62^. The K-segments are found located near the C-terminal region and supposed to be involved in the formation of class A2 amphipathic α-helix^63^. The helix is considered to establish hydrophobic interactions with other proteins in order to stabilize cell membranes^64^. Thus, the role of the K-segment may be hydropobic interaction with partially denatured proteins and protect the cell membranes, especially under the stressed condition^65^,^66^. Another important S-segment (a tract of Ser-residues) is the motif S in our analysis and it has been demonstrated that Ser-residues in the segment can be phosphorylated and leads to calcium binding^67^. The phosphorylation is also related to the binding of nuclear localization signal peptides to nuclear transport^65^. These motifs repetitions are variable in *dehydrin* proteins, especially in gymnosperms compared to the angiosperms. The *dehydrin-5* lack the motif S, *dehydrin-4* has three repetition of motif K, while *dehydrin-2* has only one motif K. A recent study also reported novel conserved segments are associated with differential expression patterns in *Pinus pinaster*^68^. In that study the discovered novel conserved A - segment (EAASYYP) and E- segment (GHGYEGQFTPEEAEQQKH) which also could be found in *dehydrin* −2/3/4/6 in our study but could not be found in *dehydrin*-5. These diversified motif structures presumably related to the divergent tolerance abilities observed but the detailed underlying mechanisms remain to be explored.

## Additional Information

**How to cite this article**: Gao, J. and Lan, T. Functional characterization of the late embryogenesis abundant (LEA) protein gene family from *Pinus tabuliformis* (Pinaceae) in *Escherichia coli*. *Sci. Rep.* **6**, 19467; doi: 10.1038/srep19467 (2016).

## Supplementary Material

Supplementary Information

Supplementary Data

## Figures and Tables

**Table 1:** The characteristics of the *P.tabuliformis* LEA proteins.

**Groups**	Pfamgroups	**Proteins**	Proteinlength(aa)	Molecularweights(kD)	Predicted subcellularlocalization
LEA1	LEA1	PtaLEA1-1	114	12.55	other
		PtaLEA1-2	117	12.51	other
LEA2	LEA2	PtaLEA2-1	190	21.40	other
LEA3	LEA3	PtaLEA3-1	113	12.22	Mitochondrion
		PtaLEA3-2	96	10.72	Mitochondrion
		PtaLEA3-3	106	11.80	Mitochondrion
		PtaLEA3-4	104	11.07	Mitochondrion
		PtaLEA3-5	103	11.31	Mitochondrion
		PtaLEA3-6	89	9.81	Mitochondrion
LEA4	LEA4	PtaLEA4-1	153	16.02	other
		PtaLEA4-2	98	10.44	other
		PtaLEA4-3	314	33.99	other
LEA5	LEA5	PtaLEA5-1	246	26.96	Chloroplast
SMP	SMP	PtaSMP-1	270	27.63	other
		PtaSMP-2	262	27.46	other
		PtaSMP-3	282	29.46	other
		PtaSMP-4	261	26.60	other
dehydrin	Dehydrin	dehydrin-1	177	18.88	other
		dehydrin-2	165	18.10	other
		dehydrin-3	238	25.85	other
		dehydrin-4	135	14.62	other
		dehydrin-5	93	10.32	other
		dehydrin-6	161	17.68	other
