# Supplementary material for: Functional characterization of the late embryogenesis abundant (LEA) protein gene family from Pinus tabuliformis (Pinaceae) in Escherichia coli
Source: Sci Rep. 2016 Jan 19;6:19467. doi: 10.1038/srep19467 (PMC4726009; doi:10.1038/srep19467)
Supplement: Supplementary Information [file srep19467-s1.pdf]

Manuscript title: Functional characterization of the late embryogenesis abundant (LEA) protein gene family from *Pinus tabuliformis* (Pinaceae) in *Escherichia coli*

Author: Jie Gao[1*] and Ting Lan[2]

[1]Key Laboratory of Tropical Forest Ecology, Xishuangbanna Tropical Botanical Garden, Chinese Academy of Sciences, Menglun, Yunnan, China

[2]State Key Laboratory of Systematic and Evolutionary Botany, Institute of Botany, Chinese Academy of Sciences, Beijing 10093, China [*]Corresponding to Dr. Jie Gao Phone: +86-691-8713066;

E-mail: gaojie@xtbg.org.cn

Supporting information

Supplemental Figure. 1 Weblogo illustration of the conserved motif sequences in *P. tabuliformis* genes.

Supplemental Figure. 2 Growth performance of *E.coli* transformants for pTWIN-LEA and pTWIN constructs under salt treatment. BP, BL21 cells with TWIN1 vector transformed.

Supplemental Figure. 3 Growth performance of *E.coli* transformants for TWIN-LEA and TWIN constructs under heat treatment. BP, BL21 cells with TWIN1 vector transformed.

Supplemental Table 1. PCR primers used to clone and expression of *LEA* genes in *P. tabuliformis*.

Supplemental Table 2. RT-PCR primers used to detect the expression of *LEA* genes.

Supplemental Table 3. EX-PCR primers used to expression the *P. tabuliformis LEA* genes.

Supplemental file 4. The identified 43 protein sequences of *P. taeda*.

motif 1

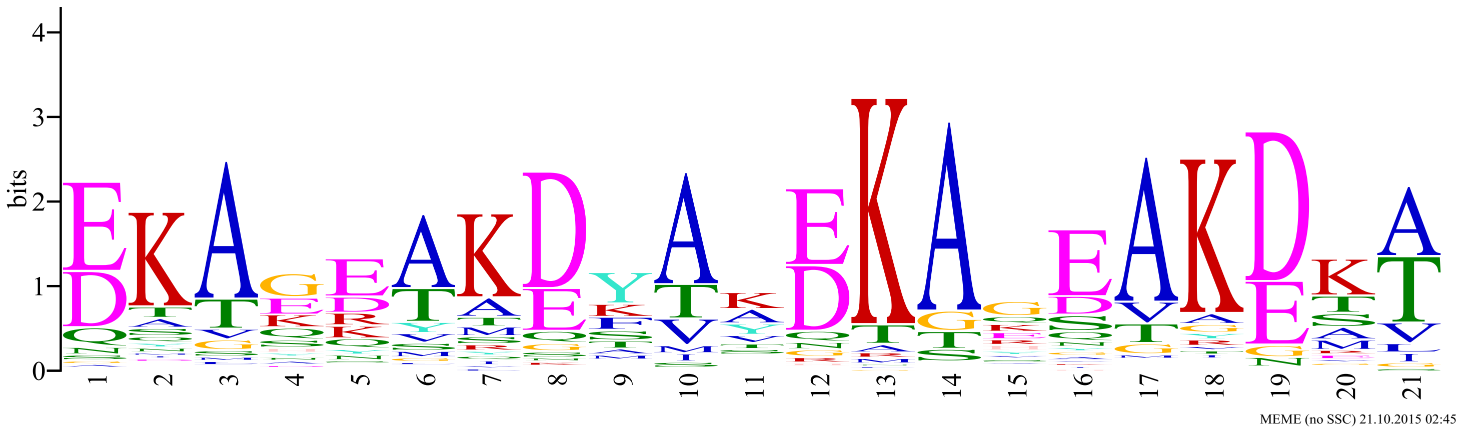

motif 2

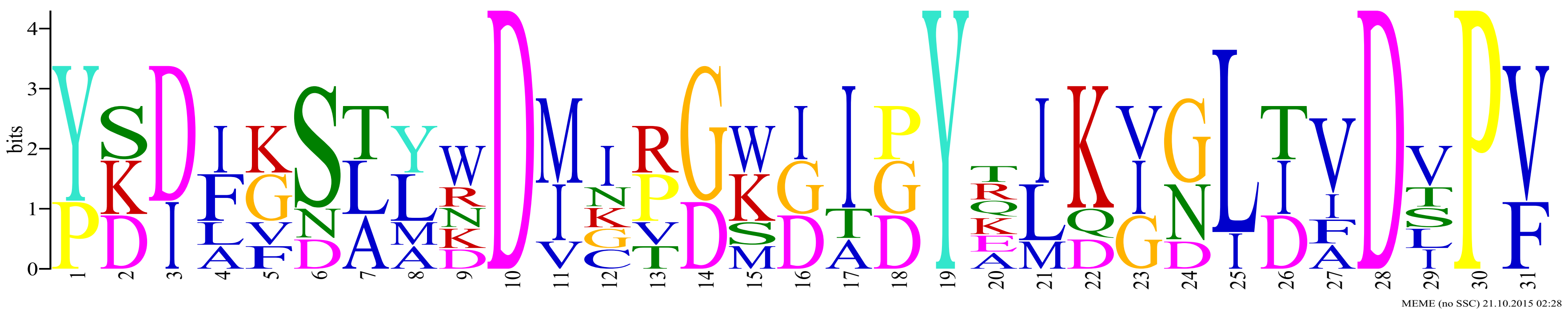

motif 3

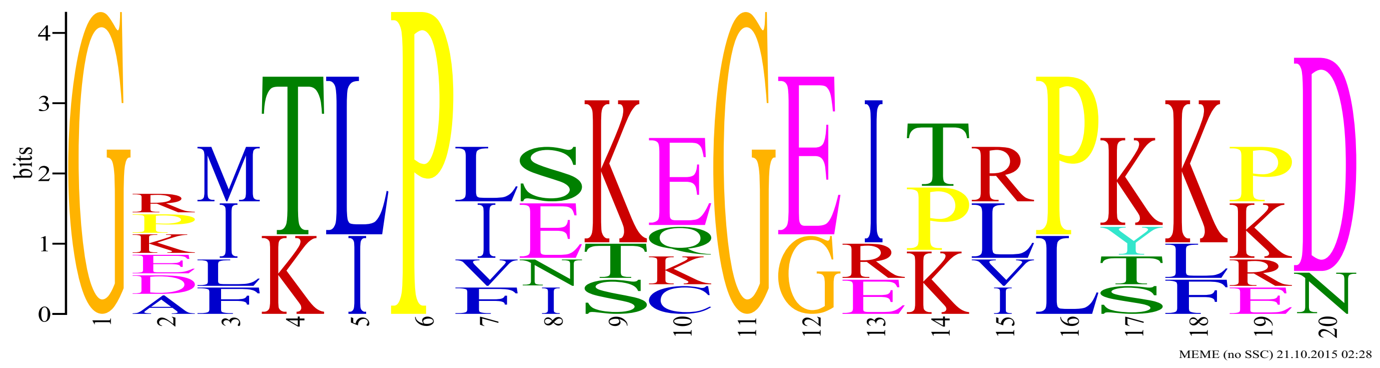

motif 4

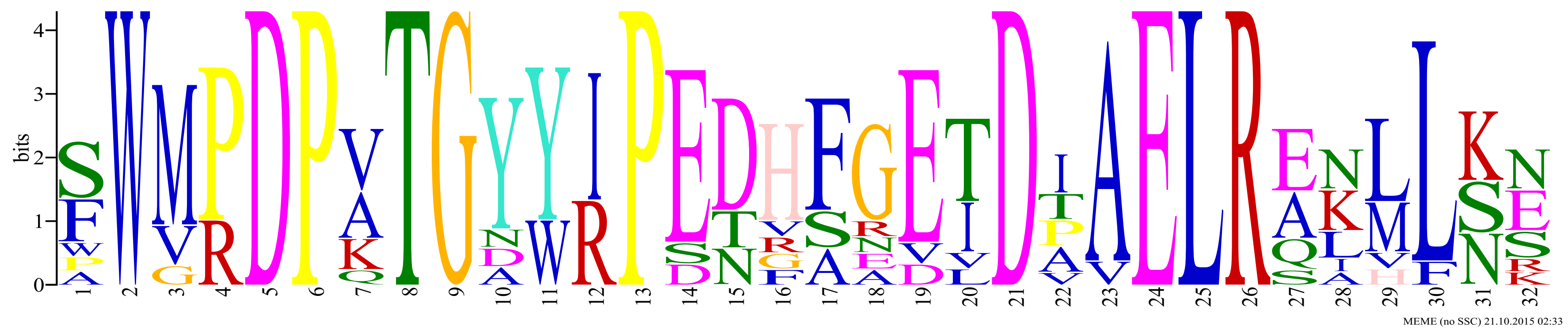

motif 5

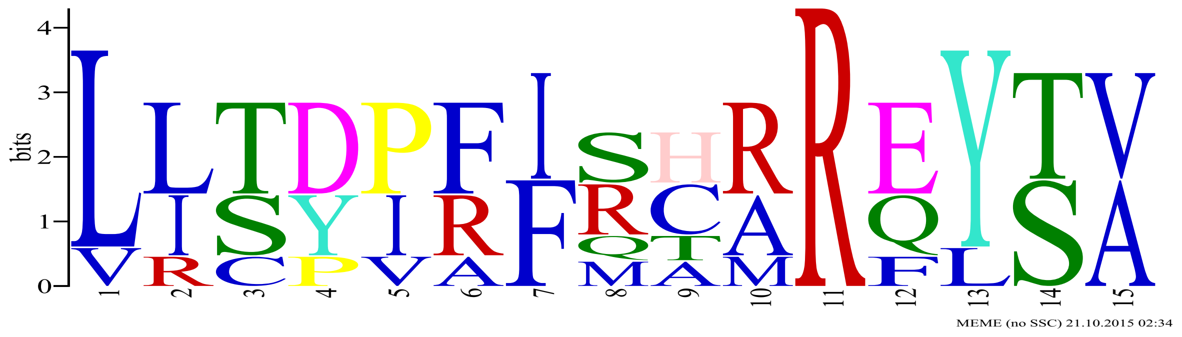

motif 6

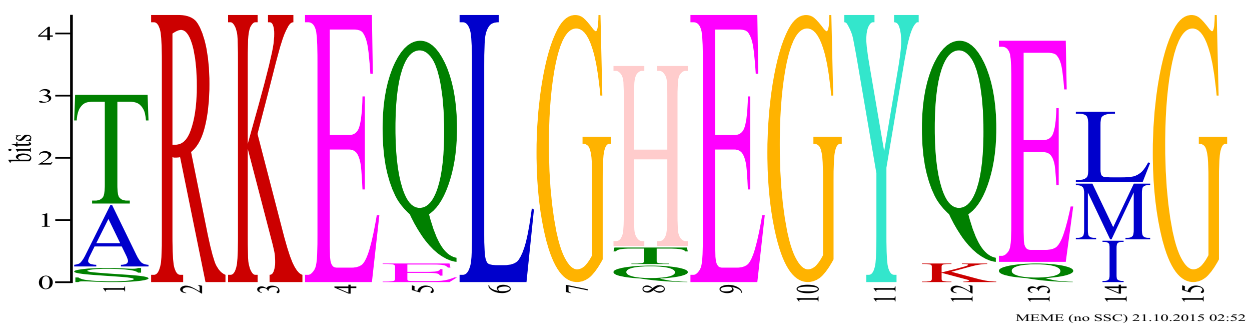

motif S

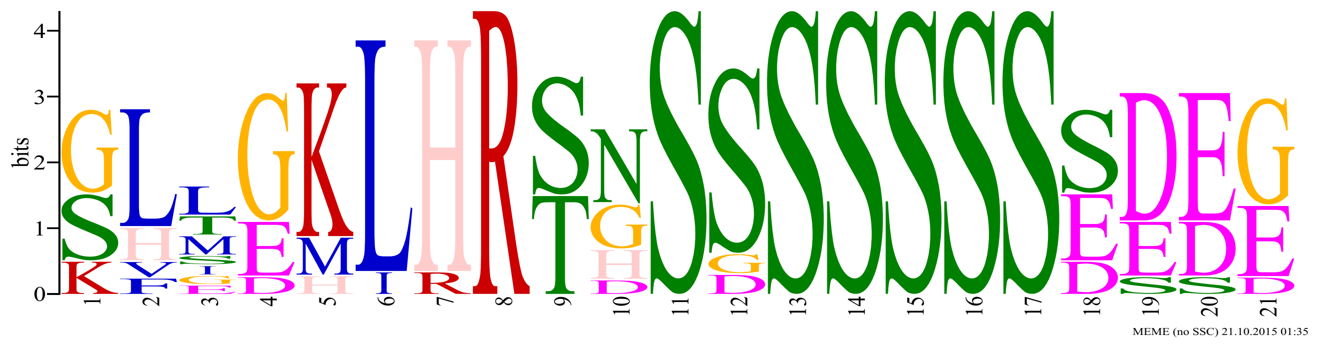

motif K

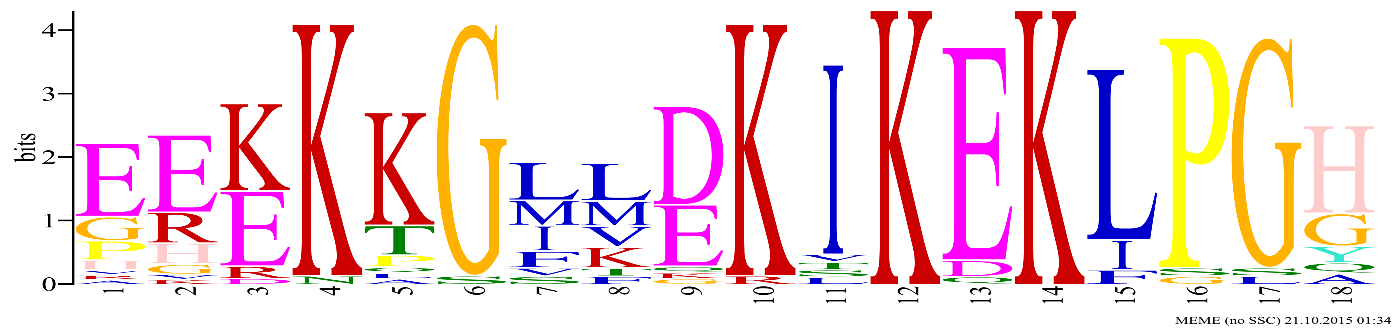

motif 9

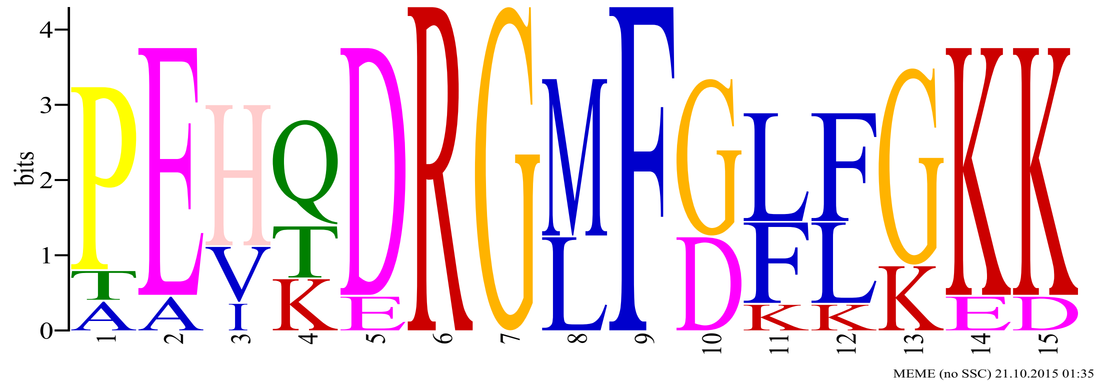

motif 10

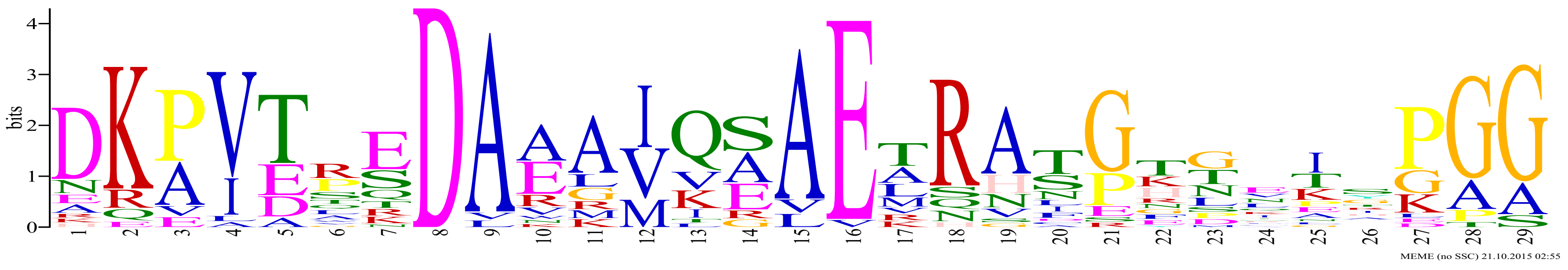

motif 11

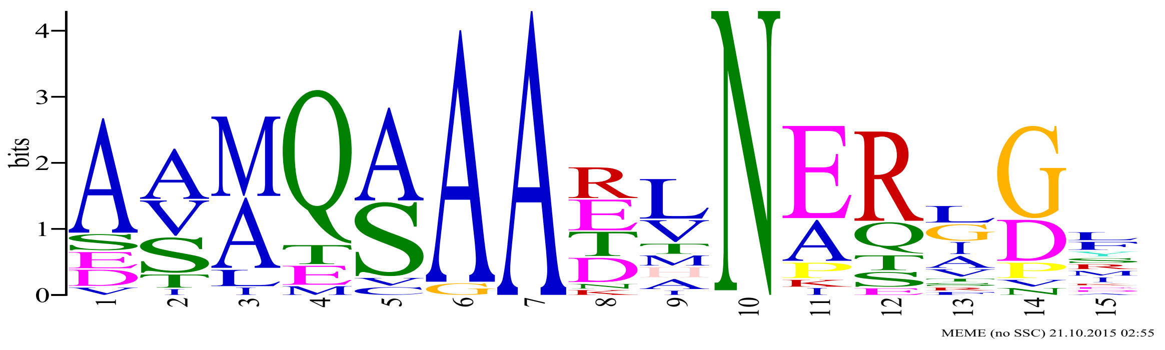

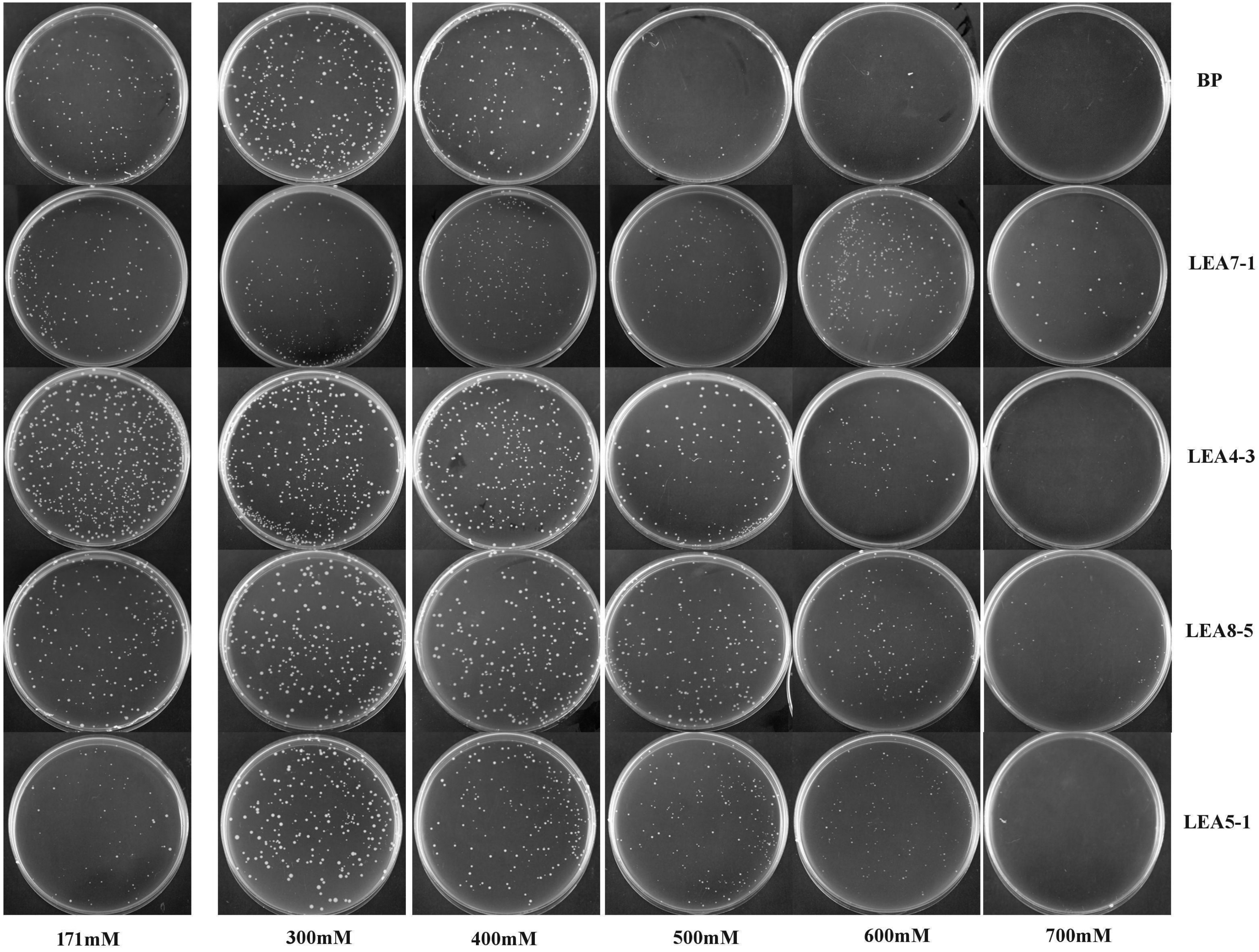
BP
LEA7-1
LEA4-3
LEA8-5
LEA5-1
171mM
300mM
400mM
500mM
600mM
700mM

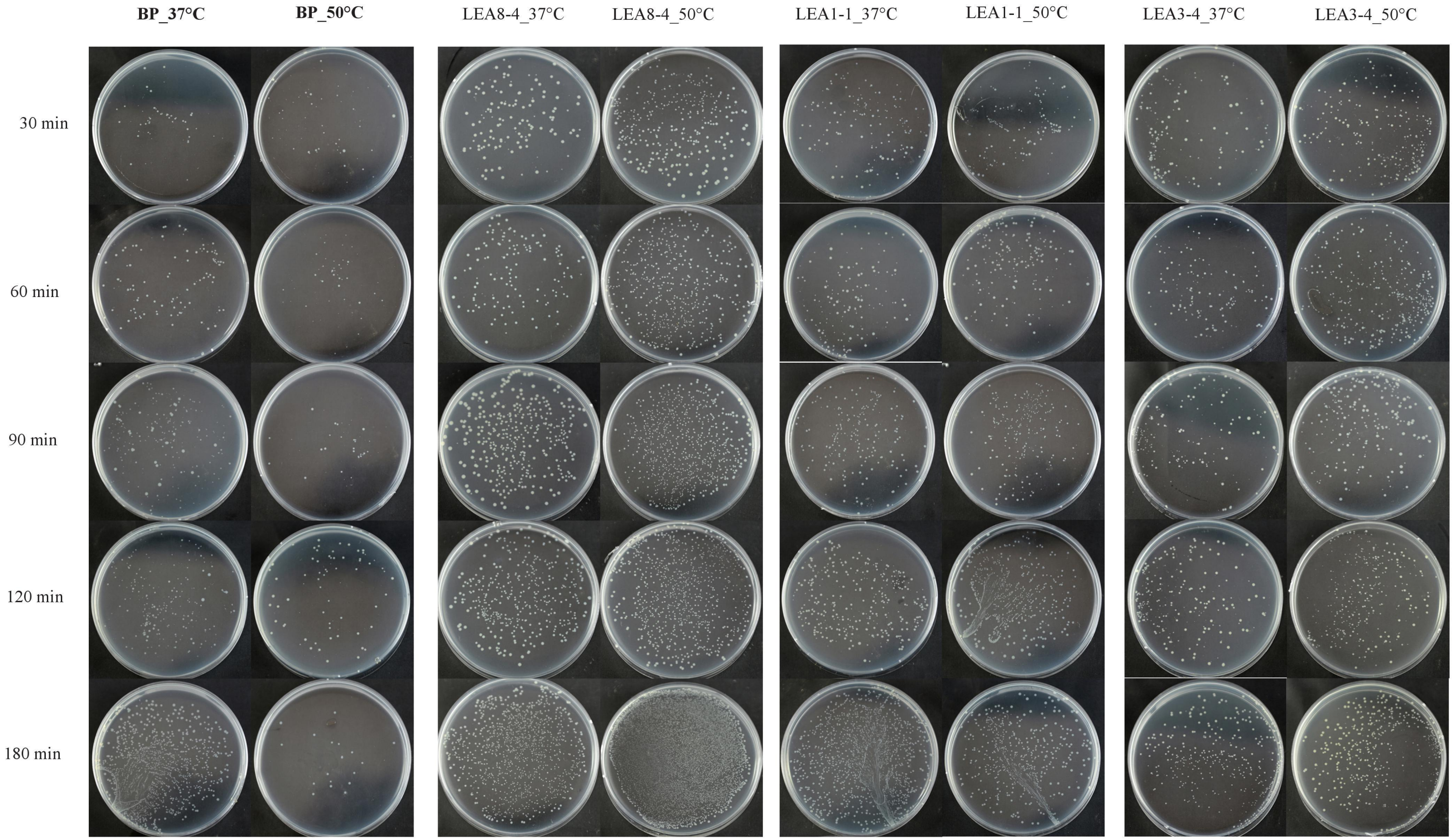
BP_37°C
BP_50°C
LEA8-4_37°C
LEA8-4_50°C
LEA1-1_37°C
LEA1-1_50°C
LEA3-4_37°C
LEA3-4_50°C
30 min
60 min
90 min
120 min
180 min

Supplemental Table1. Primers used to clone the *LEA* genes.

Genes	Primer name	Sequence (5'-3')
PtaLEA1-1	*PtaRT-LEA1-1*	GCAGCCAAGGAGAAGATGAGC
	PtaRT-LEA1-1	GGGATAAGTGGTAGTGTGAGTAGGG
PtaLEA1-2	*PtaRT-LEA1-2*	AGCGATGTGAAGGAAGGGAT
	PtaRT-LEA1-2	GTAAGTTCCAGGAGTACCAACATTC
PtaLEA2-1	*PtaRT-LEA2-1*	TATCAAGCCCGGAAGCATTATC
	PtaRT-LEA2-1	ATGGGTCCAAATGGAGAGTCAAC
PtaLEA3-1	*PtaRT-LEA3-1*	ATGGCCAGATCTCTGCCC
	PtaRT-LEA3-1	TGCTACATCAGTTACCCCAAAA
PtaLEA3-2	*PtaRT-LEA3-2*	ATAATTTTCAATGGCTGCCG
	PtaRT-LEA3-2	AATTGAGAAATTTTACATCAATTCA
PtaLEA3-3	*PtaRT-LEA3-3*	TCTACATATGAAGGTTGTGGCTG
	PtaRT-LEA3-3	CAGAAATTTTAAATCGTGTCGTT
PtaLEA3-4	*PtaRT-LEA3-4*	CTCCGAATAAGTTATCTTCTCTCAG
	PtaRT-LEA3-4	TCGGAGTTCCGCAATGTC
PtaLEA3-5	*PtaRT-LEA3-5*	TGGTCTTGTGTCCCGTTTTC
	PtaRT-LEA3-5	TTCTGGAGGAGAGCAGCTTC
PtaLEA3-6	*PtaRT-LEA3-6*	ATGGCAGGACGGCTTCTAT
	PtaRT-LEA3-6	CTACTTTCTAGATAGGAGCTTCTGC
PtaLEA4-1	*PtaRT-LEA4-1*	CGGACCAGTACAATCGGG
	PtaRT-LEA4-1	CCCAAGGGATGCTTCAGAG
PtaLEA4-2	*PtaRT-LEA4-2*	CAGTGCAAGCAATATGGGC
	PtaRT-LEA4-2	CCAAAGGGTTTCTTGACAGC
PtaLEA4-3	*PtaRT-LEA4-3*	GAGGCAGAAGAGAGACGGG
	PtaRT-LEA4-3	GGACTCTTGGGCTTTTTCTTG
PtaLEA5-1	*PtaRT-LEA5-1*	CTAAATCGTTGGGAGTGACCA
	PtaRT-LEA5-1	CCGTTCTTCTTTTCTTACTTCTCC
PtaLEA7-1	*PtaRT-LEA7-1*	ATGAAGCGCAAGATCAATTG
	PtaRT-LEA7-1	AGGCAGGTCTTCGGTAGCAGTA
PtaLEA7-2	*PtaRT-LEA7-2*	TATTTTTCAGGGTTTCATACGAC
	PtaRT-LEA7-2	CCTCCTTTTTGAGTGACGG
PtaLEA7-3	*PtaRT-LEA7-3*	AGACTCAGAAGGGTGGTGCT
	PtaRT-LEA7-3	TGGTGTTGGAAACCCATAGC
PtaLEA7-4	*PtaRT-LEA7-4*	AACGTCCTTACGGAAAGCG
	PtaRT-LEA7-4	CCACGAGCAGGTCTTCAGTAG
PtaLEA8-1	*PtaRT-LEA8-1*	CATCTGCAGAGACTGTGCCT
	PtaRT-LEA8-1	CCTTCATATCCATGCCCAT
PtaLEA8-2	*PtaRT-LEA8-2*	CAATCTGATCACATTGTCCAGAC
	PtaRT-LEA8-2	GGAATACTGCTTTCCATCCTC
PtaLEA8-3	*PtaRT-LEA8-3*	GGAGTCGAACACGGGCAT
	PtaRT-LEA8-3	CTTTGATTTTACCAAGCACACC
PtaLEA8-4	*PtaRT-LEA8-4*	ATGGCTGAACAAGCTAAGCAA
	PtaRT-LEA8-4	CGCCTTCTTCTCCTCTTCAC

PtaLEA8-5	*PtaRT-LEA8-5*	ATGGCGCAACCAGCACAC
	PtaRT-LEA8-5	TTACTCCACGTCATTCTTATTTTGT
PtaLEA8-6	*PtaRT-LEA8-6*	GAAAGCATTCTGATGAGATTGG
	PtaRT-LEA8-6	TTATTTTATCAAGCATACCCGT

Supplemental Table 2. RT-PCR primers used to detect the expression of *LEA* genes.

Genes	Primer name	Sequence (5'-3')
PtaLEA1-1	*PtaRT-LEA1-1*	GCAGCCAAGGAGAAGATGAGC
	PtaRT-LEA1-1	GGGATAAGTGGTAGTGTGAGTAGGG
PtaLEA1-2	*PtaRT-LEA1-2*	AGCGATGTGAAGGAAGGGAT
	PtaRT-LEA1-2	GTAAGTTCCAGGAGTACCAACATTC
PtaLEA2-1	*PtaRT-LEA2-1*	TATCAAGCCCGGAAGCATTATC
	PtaRT-LEA2-1	ATGGGTCCAAATGGAGAGTCAAC
PtaLEA3-1	*PtaRT-LEA3-1*	ATGGCCAGATCTCTGCCC
	PtaRT-LEA3-1	TGCTACATCAGTTACCCCAAAA
PtaLEA3-2	*PtaRT-LEA3-2*	ATAATTTTCAATGGCTGCCG
	PtaRT-LEA3-2	AATTGAGAAATTTTACATCAATTCA
PtaLEA3-3	*PtaRT-LEA3-3*	TCTACATATGAAGGTTGTGGCTG
	PtaRT-LEA3-3	CAGAAATTTTAAATCGTGTCGTT
PtaLEA3-4	*PtaRT-LEA3-4*	CTCCGAATAAGTTATCTTCTCTCAG
	PtaRT-LEA3-4	TCGGAGTTCCGCAATGTC
PtaLEA3-5	*PtaRT-LEA3-5*	TGGTCTTGTGTCCCGTTTTC
	PtaRT-LEA3-5	TTCTGGAGGAGAGCAGCTTC
PtaLEA3-6	*PtaRT-LEA3-6*	ATGGCAGGACGGCTTCTAT
	PtaRT-LEA3-6	CTACTTTCTAGATAGGAGCTTCTGC
PtaLEA4-1	*PtaRT-LEA4-1*	CGGACCAGTACAATCGGG
	PtaRT-LEA4-1	CCCAAGGGATGCTTCAGAG
PtaLEA4-2	*PtaRT-LEA4-2*	CAGTGCAAGCAATATGGGC
	PtaRT-LEA4-2	CCAAAGGGTTTCTTGACAGC
PtaLEA4-3	*PtaRT-LEA4-3*	GAGGCAGAAGAGAGACGGG
	PtaRT-LEA4-3	GGACTCTTGGGCTTTTTCTTG
PtaLEA5-1	*PtaRT-LEA5-1*	CTAAATCGTTGGGAGTGACCA
	PtaRT-LEA5-1	CCGTTCTTCTTTTCTTACTTCTCC
PtaLEA7-1	*PtaRT-LEA7-1*	ATGAAGCGCAAGATCAATTG
	PtaRT-LEA7-1	AGGCAGGTCTTCGGTAGCAGTA
PtaLEA7-2	*PtaRT-LEA7-2*	TATTTTTCAGGGTTTCATACGAC
	PtaRT-LEA7-2	CCTCCTTTTTGAGTGACGG
PtaLEA7-3	*PtaRT-LEA7-3*	AGACTCAGAAGGGTGGTGCT
	PtaRT-LEA7-3	TGGTGTTGGAAACCCATAGC
PtaLEA7-4	*PtaRT-LEA7-4*	AACGTCCTTACGGAAAGCG
	PtaRT-LEA7-4	CCACGAGCAGGTCTTCAGTAG
PtaLEA8-1	*PtaRT-LEA8-1*	CATCTGCAGAGACTGTGCCT
	PtaRT-LEA8-1	CCTTCATATCCATGCCCAT
PtaLEA8-2	*PtaRT-LEA8-2*	CAATCTGATCACATTGTCCAGAC
	PtaRT-LEA8-2	GGAATACTGCTTTCCATCCTC
PtaLEA8-3	*PtaRT-LEA8-3*	GGAGTCGAACACGGGCAT
	PtaRT-LEA8-3	CTTTGATTTTACCAAGCACACC
PtaLEA8-4	*PtaRT-LEA8-4*	ATGGCTGAACAAGCTAAGCAA
	PtaRT-LEA8-4	CGCCTTCTTCTCCTCTTCAC

PtaLEA8-5	*PtaRT-LEA8-5*	ATGGCGCAACCAGCACAC
	PtaRT-LEA8-5	TTACTCCACGTCATTCTTATTTTGT
PtaLEA8-6	*PtaRT-LEA8-6*	GAAAGCATTCTGATGAGATTGG
	PtaRT-LEA8-6	TTATTTTATCAAGCATACCCGT

Supplemental Table 3. EX-PCR primers used to expression the *P.tabliufrims LEA* genes.

Genes	Primer name	Sequence (5'-3')
PtaLEA1-1	*PtaEX-LEA1-1*	A***GCGGCCGC***ATGCAACCTGTAAAGAATGCA
	PtaEX-LEA1-1	T***TACTAG***TTATCATAAGTACTTGCCGGGAG
PtaLEA1-2	*PtaEX-LEA1-2*	A***GCGGCCGC***ATGCAGACGGCCAAGGAG
	PtaEX-LEA1-2	T***TACTAG***TTATCACAGGTAATCTCTACCAGTGTTG
PtaLEA2-1	*PtaEX-LEA2-1*	A***GCGGCCGC***ATGACAATAATCTACGATGATATC
	PtaEX-LEA2-1	T***TACTAG***TTATCAATACTCATCGTCCTCG
PtaLEA3-2	*PtaEX-LEA3-2*	A***GCGGCCGC***ATGGCTGCCGGTGCTCTG
	PtaEX-LEA3-2	T***TACTAG***TTAATTTGGTCCTCCGCGAGA
PtaLEA3-4	*PtaEX-LEA3-4*	A***GCGGCCGC***ATGGCAAAGCTTGTGTCTCC
	PtaEX-LEA3-4	T***TACTAG***TTATCAGGAGGAGAGGACCTTCG
PtaLEA4-2	*PtaEX-LEA4-2*	A***GCGGCCGC***ATGGCGGATCAGTGCAAG
	PtaEX-LEA4-2	T***TACTAG***TTACTACTGATCGCTACCACCAAAG
PtaLEA4-3	*PtaEX-LEA4-3*	A***GCGGCCGC***ATGGCTTCAGAACAGGAAAAC
	PtaEX-LEA4-3	T***TACTAG***TTATCACCCACGTCGTTGAAAG
PtaLEA5-1	*PtaEX-LEA5-1*	A***GCGGCCGC***ATGGAGGGATTGATAATGAAAAT
	PtaEX-LEA5-1	T***TACTAG***TTAGTAGCCAAGCCTTTCCTTA
PtaLEA7-1	*PtaEX-LEA7-1*	A***GCGGCCGC***ATGAATCAGGGGCAGGCG
	PtaEX-LEA7-1	T***TACTAG*****TTA**TCATGTCCGAAACCCCATG
PtaLEA7-3	*PtaEX-LEA7-3*	A***GCGGCCGC***ATGCAGAGTGCGGAGGCA
	PtaEX-LEA7-3	T***TACTAG***TTACTTCTCCGAGTGGCTGCT
PtaLEA8-2	*PtaEX-LEA8-2*	A***GCGGCCGC***ATGGCGGAAGAAGCACCAG
	PtaEX-LEA8-2	T***TACTAG***TTACTGTTCCCCATCATTCTTGTTAT
PtaLEA8-4	*PtaEX-LEA8-4*	A***GCGGCCGC***ATGGCTGAACAAGCTAAGCA
	PtaEX-LEA8-4	T***TACTAG***TTACTAGTGTCCAGGCAGCTTCTC
PtaLEA8-5	*PtaEX-LEA8-5*	A***GCGGCCGC***ATGGCGCAACCAGCACAC
	PtaEX-LEA8-5	T***TACTAG***TTACTCCACGTCATTCTTATTTTGTT
